# Preparation and Evaluation of Amorphous Solid Dispersions for Enhancing Luteolin’s Solubility in Simulated Saliva

**DOI:** 10.3390/polym15010169

**Published:** 2022-12-29

**Authors:** Maria Koromili, Afroditi Kapourani, Panagiotis Barmpalexis

**Affiliations:** 1 Laboratory of Pharmaceutical Technology, Division of Pharmaceutical Technology, School of Pharmacy, Faculty of Health Sciences, Aristotle University of Thessaloniki, 541 24 Thessaloniki, Greece; 2 Natural Products Research Centre of Excellence-AUTH (NatPro-AUTH), Center for Interdisciplinary Research and Innovation (CIRI-AUTH), 57001 Thessaloniki, Greece

**Keywords:** luteolin, amorphous solid dispersions, solubility, simulated saliva, oral cavity, periodontitis

## Abstract

Luteolin (LUT), a bioactive flavonoid, possesses various pharmacological properties, including antioxidant, antimicrobial, anti-allergic, cardio-protective, and anti-cancer activity. Among them, LUT’s administration for the treatment of periodontal disease is very promising. However, its low water solubility magnifies the challenge of formulating LUT into an effective dosage form. In this vein, the aim of the present study examines the preparation of amorphous solid dispersions (ASD) for the solubility improvement of LUT in saliva. At first, the physicochemical properties of the active pharmaceutical ingredient (API) were studied before the selection of the most suitable ASD matrix/carrier. For this reason, six commonly used polymeric ASD matrix/carriers (namely, povidone, PVP; copovidone, coPVP; hydroxypropyl cellulose, HPC-SL; hydroxypropyl methyl cellulose acetate succinate, HPMC-AS; Eudragit^®^ RS, Eud-RS; and Soluplus^®^, SOL) were screened via the film casting method, as to whether they could suspend the drug’s recrystallization. The most promising matrix/carriers were then evaluated, based on their ability to inhibit LUT’s precipitation after its solubilization, via the solvent shift method. Based on both screening methods, it was determined that PVP was the most promising matrix/carrier for the preparation of LUT’s ASDs. Hence, in a further step, after the successful testing of components’ miscibility, LUT-PVP ASDs were prepared via the solvent evaporation method. These systems (examined via powder X-ray diffractometry, pXRD) showed full API amorphization immediately after preparation and excellent physical stability (since they were stable after 3 months of storage). The study of LUT-PVP ASD’s ATR-FTIR (Attenuated Total Reflectance-Fourier Transform Infrared) spectra demonstrated strong H-bonds between the molecules of the drug and the matrix/carrier, while molecular dynamics (MD) simulations were able to shed light on these drug–matrix/carrier interactions, at a molecular level. Finally, in vitro dissolution studies in simulated saliva proved that the prepared ASDs were able to significantly enhance LUT’s dissolution profile. Hence, according to findings of the present work, the preparation of LUT-ASDs utilizing PVP as the polymeric matrix/carrier is regarded as a highly promising technique for the improvement of API’s solubility in the oral cavity.

## 1. Introduction

Flavonoids are one of the most important and diverse groups of phytonutrients that consist of many polyphenolic compounds that are ubiquitous in the plant kingdom [1,2,3]. Due to their numerous biological activities, such as antioxidant, anti-inflammatory, antibacterial, anti-carcinogenic, etc., flavonoids show many nutraceutical and pharmaceutical applications [4,5]. In particular, flavones represent one of the largest subclasses of flavonoids that appear to have a positive impact on human health, as a result of their various pharmacological properties [6,7]. Among them, luteolin (LUT) (3′,4′,5,7-tetrahydroxy flavonoid, Figure S1, Supplementary Materials) is an important flavone, thanks to its presence in many medicinal herbs and edible plants including broccoli, thyme, carrots, peppers, cabbage, and celery [8,9].

LUT has been widely researched for its anti-inflammatory, anti-diabetic, and anti-cancer potential, demonstrating remarkable results in several studies, especially in the prevention of the metastatic steps (invasion, colonization) in breast cancer [10,11,12,13,14,15,16,17,18,19]. In addition, numerous scientists have investigated luteolin’s antioxidant, antimicrobial, anti-allergic, and cardio-protective activities [20,21,22,23,24,25,26,27,28], while the fact that LUT crosses the blood–brain barrier (BBB), thanks to its four-phenolic group structure, demonstrates its neuroprotective action against chronic illnesses such as autism spectrum disorder and Alzheimer’s disease [29,30]. Within this concept, in the last several years, various research groups have focused their efforts on the beneficial effect of LUT on periodontal disease [31,32,33,34]. In these studies, LUT, as an anti-inflammatory factor, demonstrates noteworthy results in the prevention and treatment of periodontitis. Specifically, certain of its pathological causes, such as alveolar bone loss, osteoclastic and collagenase activity, tissue damage, and neutrophilic infiltration, were reduced by the administration of LUT. Furthermore, Quan et al. highlighted that 1 μmol/L LUT promotes osteogenic differentiation as a potential coping mechanism for periodontitis [34]. However, although the therapeutic use in the treatment of periodontal disease shows great potential, the challenge of formulating LUT into an efficient pharmaceutical form is high due to its poor aqueous solubility leading to low bioavailability.

Among other polar solvents, LUT appears to have significantly low solubility in water [35]. To circumvent this, numerous techniques have been examined, including cyclodextrins, phyto-phospholipid complexes, cocrystals, and nano-technology-based drug delivery systems (nanoparticles, zein nanoparticles, nanoemulsion, nanocrystals, etc.) [36,37,38,39,40,41,42,43,44]. Among them, the preparation of solid dispersions (either amorphous or crystalline) appeared as a promising approach [45,46]. In general, amorphous solid dispersions (ASDs), namely, the dispersion of one or more active pharmaceutical ingredients (APIs) in a hydrophilic matrix/carrier at the molecular level, are widely utilized for the successful formulation of poorly water-soluble drugs (PWSDs) [47,48,49]. The significance of this formulation technique is realized by the several widely administrated pharmaceutical products that have been on the market thanks to their ASD form (i.e., Sporanox^®^-itraconazole, Mefoxin^®^-cefoxitin, Indocin^®^-indomethacin, etc.) [50].

Returning our attention to LUT’s poor aqueous solubility, several studies have indicated that when the drug is administrated orally, its bioavailability is decreased, reaching low plasma concentrations due to the high intestinal and hepatic first-pass effects (this stands for all flavonoids) [51,52]. In this direction, to avoid first-pass metabolism, a different route of administration for periodontal patients could be considered (such as the sublingual/buccal route) [53]. However, in such cases, the extremely lower quantities of dissolution volumes available for a drug’s solubilization (compared to the stomach or the intestine) magnifies the formulation development difficulties.

Thus, the purpose of the current work is to investigate the development of ASDs as a drug delivery strategy to enhance LUT’s solubility in the saliva of the oral cavity. The present study is the first rigorous scientific evaluation of LUT’s solubility improvement using this technique, and in this sense, it tries to set the foundations for the successful sublingual/buccal or topical (in the mouth) administration of the API.

## 2. Materials and Methods

### 2.1. Materials

LUT was purchased from Santa Cruz Biotechnology (Dallas, TX, USA). PVP (Kollidon^®^K90), coPVP (Kollidon^®^VA64), and SOL (i.e., polyvinyl caprolactam-polyvinyl acetate-polyethylene glycol graft copolymer) were obtained from BASF (Ludwigshafen, Germany), while HPC-SL and HPMCAS were obtained from Shin-Etsu (Nigata, Japan). EPO was obtained from Evonik (Essen, Germany). All other reagents used in the specific study meet the analytical and pharmaceutical requirements.

### 2.2. Thermophysical Characterization of LUT

#### 2.2.1. Glass Forming Ability (GFA)

The GFA of LUT was examined by the use of a DSC heating–cooling–heating cycle, according to a method proposed by Baird et al. [54]. In particular, samples containing weighed amounts of LUT (~5.0 mg) were heated in a DSC 204 F1 Phoenix heat-flux DSC (NETZSCH, Germany) up to 360 °C, at a rate of 20 °C per minute. The samples were then kept at an isothermal state for 3 min, to erase any previous thermal history, and cooled at a rate of 20 °C per minute to 0 °C, before the second heating (at the same rate) up to 360 °C. The standard deviations of the temperatures and enthalpies determined in this study did not surpass 1.0 °C and 3.0 J/g, respectively. Nitrogen flow (50 mL/min) was implemented to achieve a constant thermal environment within the DSC cell. The instrument was calibrated for temperature using benzophenone, indium, and tin. All experiments were held in triplicate.

#### 2.2.2. Thermal Stability

In order to evaluate LUT’s stability in thermal events occurring during the ASD preparation, thermogravimetric analysis (TGA, Shimadzu TGA-50 thermogravimetric analyzer, Tokyo, Japan) was conducted. This is important, in order to clarify whether thermal processing techniques (i.e., melt mixing) are suitable for the formulation of ASDs for this API. For this purpose, the analysis began by weighing approximately 5 mg of LUT before placing this into suitable aluminum sample pans, attached to a sensitive microbalance assembly. The samples were heated from 25 °C to 360 °C at a rate of 20 °C per minute, using nitrogen as purge gas (with a flow rate of 25 mL per minute). The change in LUT’s weight was recorded concerning temperature.

### 2.3. Selection of ASD Matrix/Carrier

The screening for the most promising matrix/carrier was made via two techniques: (1) film casting and (2) solvent shift.

#### 2.3.1. Film Casting Method

Quantities of 0.1 g of the API and 0.1 g of each matrix/carrier (i.e., PVP, coPVP, HPC-SL, SOL, EPO, HPMCAS) were co-dissolved in appropriate amounts (3–5 mL) of solvent (methanol, MeOH, was used in all cases, except for PVP and coPVP where dimethyl sulfoxide, DMSO, was used). In both cases, the two solvents (i.e., MeOH and DMSO) were selected in order to fully solubilize the API. A high drug-content-to-carrier ratio (i.e., 50% *w*/*w*) was chosen to accelerate the recrystallization of the API. The prepared API-matrix/carrier solutions were placed in standard microscopy slides and rapidly dried in a vacuum oven at 40 °C (3 mbar) for 3 h to form a thin film. In terms of the systems LUT-PVP and LUT–coPVP, where DMSO was used for the co-dissolution, the microscopy slides were dried in a vacuum oven at 75 °C (3 mbar) for a further 5 h in order to completely remove the organic solvent (verified by TGA analysis). Microscopy slides, containing only the neat LUT, were also examined for comparison reasons. After the solvent’s elimination, the formation of an amorphous dispersion was confirmed via polarized light microscopy (PLM) on an Olympus BX41 polarized light microscope (Olympus, Tokyo, Japan). The samples were then stored in desiccators for several days at high temperature (40 °C) and relative humidity (75%). During the conduction of the study, microscopic slides were investigated for API recrystallization using PLM (birefringence under crossed polars) at predetermined time intervals. At the end of the observations with the specific methodology, the most comparatively suitable matrix/carrier was selected.

#### 2.3.2. Solvent Shift Measurements

The capacity of the matrix/carriers to inhibit LUT’s precipitation after its solubilization was also evaluated. For this purpose, solvent shift measurements were conducted in simulated saliva (pH 6.8, using 8.00 g/L NaCl, 0.19 g/L KH2PO4, 2.38 g/L Na_2_HPO_4_), based on a modified method adopted from a previous study [55]. In particular, 5 mL of LUT solution in DMSO (10%, *w*/*v*) was gradually added to the dissolution medium containing the simulated saliva (with or without the polymer). The polymer concentration was constant at 0.1 % *w*/*w* in all cases. All experiments were performed in a USP II (paddles) dissolution tester (PT-DT70, PharmaTest, Turku, Finland) at 37 °C ± 0.5 °C and 100 rpm. After predetermined time intervals (i.e., 5, 15, 30, 45, 60, 90, 120, and 240 min), aliquots of 3 mL were removed, passed through a 0.10 μm membrane filter (Millipore Millex-HV, Burlington, MA, USA), and diluted with MeOH to eliminate the risk of drug crystallization after the sampling. LUT’s concentration was determined spectrophotometrically at 349 nm.

### 2.4. Miscibility Evaluation

A critical parameter of the successful formation of an ASD system is the total miscibility of components (i.e., the API and the matrix/carrier). Therefore, the miscibility of LUT with the selected matrix/carrier (derived from the combination of film casting and solvent shift methods) was thoroughly examined with the following methods:

#### 2.4.1. Estimation of the Flory–Huggins (FH) Interaction Parameter

Monitoring the changes in the melting temperature (*T_m_*), melt endothermic peaks, and heat of fusion (Δ*H_fus_*) of the drug was used to evaluate the miscibility of the components. The existence of miscibility is verified by a decrease in *T_m_* and Δ*H_fus_* with increasing polymer concentration. More specifically, this *T_m_* depression is related to the Flory–Huggins (FH) interaction parameter (*χ*) based on Formula (1).

(1/(2) − 1/*T_m_*(1)) = −*R*/Δ*H_fus_* [*l*_(1)_ + (1 − 1/*m*) *Φ*_(2)_ + *χΦ*^2^_(2)_] (1)

where *T_m_*(1) and *T_m_*(2) are the melting temperatures of the pure LUT and LUT in the presence of a suitable matrix/carrier (polymer), respectively; *R* is the gas constant; Δ*H_fus_* refers to the heat of fusion of the pure drug; m is the ratio by volume of polymer to LUT; *χ* is the FH interaction parameter; and *Φ*_(1)_ and *Φ*_(2)_ are the volume fractions of LUT and the selected matrix/carrier, respectively. To estimate the interaction parameter, *χ*, the slope of the *Φ*_(1)_ to (1/*T_m_*(2) − 1/*T_m_*(1)) ∗ (Δ*H_fus_*/−*R*) − [*ln*Φ_(1)_ + (1 − 1/*m*) Φ_(2)_ + *χΦ*^2^_(2)_] graph is used.

#### 2.4.2. Miscibility Evaluation via Solubility Parameter (δ)

In addition to the FH parameter, Hansen solubility parameters (calculated based on the Hoftyzer–Van Krevelen group contribution method) were measured to determine the miscibility of LUT and the matrix/carrier [56]. In this direction, the total solubility parameter (δ_t_) is calculated depending on the molar volume (V) and the dispersion (d), polar (p), and hydrogen bonding (h) forces based on the following Equation (2):

(2) $$\delta_{t}=\sqrt{\delta_{d}^{2}+\delta_{p}^{2}+\delta_{h}^{2}}$$

where, *δ_d_*, *δ_p_*, and *δ_h_* are the partial solubility parameters for intermolecular dispersion, and polar and hydrogen bonding forces, respectively. Based on Greenhalgh et al. [57], API-polymer blends are assumed to be immiscible at Δδ_t_ > 10 MPa^1/2^, miscible at Δδ_t_ <7 MPa^1/2^, and likely to form a glassy solid solution at Δδ_t_ less than 2 MPa^1/2^.

#### 2.4.3. Miscibility Evaluation via Differential Scanning Calorimetry (DSC)

In addition to the theoretical approaches, the miscibility of the studied system was experimentally evaluated via DSC. A Netzsch DSC 204 F1 Phoenix heat flux (NETZSCH, Germany) was used. In brief, the neat API, the selected matrix/carrier, and the physical mixtures of LUT with the matrix/carrier (using 10, 20, and 30% *w*/*w* of the API) were subjected to a cyclic (heat–cool–heat) DSC scanning procedure. Then, 5.0 mg of each sample were sealed in suitable aluminum pans and were heated from 0 °C to 360 °C (at a rate of 10 °C per minute) until the complete melting of the API. The samples were kept there for 2 min, to delete their thermal history, and then rapidly cooled at 0 °C and reheated at 360 °C at the same heating rate. All thermograms used the NETZSCH Proteus—Thermal Analysis software package version 5.2.1 (NETZSCH, Waldkraiburg, Germany). Each experiment was performed three times.

### 2.5. Preparation of LUT-Loaded ASDs

The preparation of amorphous LUT and ASDs (using the most suitable matrix/carrier) was conducted via solvent evaporation. In brief, appropriate amounts of the drug and the most promising matrix/carrier were co-dissolved, using DMSO as a solvent (5–8 mL). The prepared API-matrix/carrier solutions were dried in a vacuum oven at 75 °C (3 mbar) for 8 h, in order to completely eliminate any remaining solvent. The final ASDs were hermetically sealed in amber glass vials using aluminum crimp caps and placed in desiccators prior to subsequent use. The homogeneity of LUT within the prepared ASDs was evaluated spectrophotometrically at 349 nm by testing three randomly selected samples. LUT’s concentration was found to be between 98.0–101.0%, while the % relative standard deviation (%RSD) was less than 1.0% in all cases.

### 2.6. Evaluation of LUT-Loaded ASDs

#### 2.6.1. Physical State

The physical state of the API within the prepared ASD samples was evaluated via powder X-ray diffractometry (pXRD). To investigate the crystallinity of the raw materials and the ASD systems, a Bruker XRD-diffractometer (D2 Phaser, Bruker, Karlsruhe, Germany) with a CuKα radiation (λ = 0.15405 nm for CuKα) was used. All samples were scanned from 5 to 45°, 2 <theta>.

#### 2.6.2. Molecular Interactions

The molecular interactions between the drug and the selected matrix/carrier were evaluated by ATR-FTIR spectroscopy and molecular dynamics (MD) simulations.

##### ATR-FTIR Spectroscopy

During ATR-FTIR analysis, the physical mixtures of the API and the selected matrix/carrier, the developed ASD, and the initial raw material were examined. For every spectrum, 64 successive scans were received in the region of 750–4000 cm^−1^ (at a resolution of 4 cm^−1^), and from the average of these measurements, the final spectrum was obtained. All spectra were recorded with the use of Shimadzu IR-Prestige-21-FT-IR infrared spectrometer (Tokyo, Japan) coupled with a horizontal Golden Gate MKII single-reflection ATR system (Specac, Kent, UK) equipped with ZnSe lenses after appropriate background subtraction. All spectra analysis was made using the IRsolution 1.30 software (Shimadzu, Tokyo, Japan).

##### Molecular Dynamics (MD) Simulations

Initial structures: The initial molecular structure of LUT was obtained from the Cambridge Structural Database (CSD deposition number: 217463) based on the published work of Cox et al. [58]. The molecular structure of the selected matrix/carrier was prepared using XenoView v.3.7.9.0 PC software (available online at http://www.vemmer.org/xenoview/xenoview.html (accessed on 15 July 2022)). In both cases, the initial structures were subjected to energy minimization using the pcff_d force field with XenoView. The steepest descent algorithm, with a tolerance limit at 1.00 × 10^−4^ kcal/mol and 10 Å maximum displacement per iteration, was used for this task. In summary, 10 independent structures were prepared and those with the lowest energy (one for each component) were chosen for further processing.

Amorphous cell construction: All amorphous assemblies were constructed using XenoView’s amorphous builder. A multistep equilibration protocol was adopted in order to prepare well-mixed and fully relaxed simulation cells [59]. The validation of the amorphous assemblies was performed by comparing the T_g_ estimated from the MD simulations (calculated by plotting the averaged specific volume, v, against temperature after heating the assemblies and subsequently cooling them within 15 equally distributed steps from 803 to 203 K) and the experimental Tg determined by DSC (using the same equipment and methodology described in Section 2.4.3).

Long-run MD simulations: The final structures were subjected to long-run (10.0 ns) MD simulations using the NPT ensemble at 1 atm and 25 °C with a cutoff radius of 10 Å, spline distance of 1 Å, Berendsen thermostat and barostat, and time step of 1 fs. Van Gunsteren and Mark criteria in regard to model development, force field selection, sampling scheme setup, and software selection/use were adopted [60].

Trajectory analysis: For assessing the formation of molecular interactions, the trajectory of the final 5 ns from the long-run MD simulation was analyzed and the radial distribution function, *g*(*r*), was calculated based on the following equation:

(3) $$g\left(r\right)=\frac{\langle V\Sigma_{i\neq j}\delta (r-\left|r_{Ai}-r_{Bi}\right|)\rangle }{\left({Ν}_{A}N_{B}-N_{AB}\right)4\pi r^{2}dr}$$

where *A* and *B* are specific atoms; *V* is the system volume; *N_A_* and *N_B_* are the particle number of atoms *A* and *B*, respectively; *N_AB_* are the number of particles belonging to atom *A* and atom *B* simultaneously; and *r_Ai_* and *r_Bj_* are the positions of particle *i* of atom *A* and particle *j* of atom *B*, respectively.

### 2.7. In Vitro Drug Release Studies in Simulated Saliva

In vitro drug release studies were conducted in simulated saliva, based on a previously published method [61]. Briefly, supersaturation studies were performed in small-scale glass vials (1.5 mL maximum volume) under stirring. The temperature was maintained at 37 °C with the use of a water bath. At predetermined time intervals (i.e., 1, 2, 5, 10, 15, and 30 min), the entire content of the dissolution vial was collected and centrifuged for 15 min. The solution fractions, free from any solid content, were then analyzed via a UV-Vis spectrometer (Pharma Spec UV-1700, Shimadzu Europa GmbH, Duisburg, Germany) at λ = 349 nm. All tests were performed in triplicate.

An equilibrium solubility study of LUT was performed in triplicate by adding an excess amount of LUT in 10 mL simulated saliva. The solution was stirred at 100 rpm at 37 °C for 24 h and the sample was then filtered and analyzed spectrophotometrically at 349 nm. LUT’s equilibrium solubility, *Cs*, equals 5.7 μg/mL.

## 3. Results and Discussion

Before the selection of the most suitable matrix/carrier and the preparation of LUT-loaded ASDs, it is important to clarify API’s GFA and its thermal stability profile, as these data determine the selection of the most appropriate formulation strategy (e.g., solvent evaporation, melt mixing, etc.).

### 3.1. Thermophysical Characterization of LUT

#### 3.1.1. GFA of LUT

**Figure 1:**
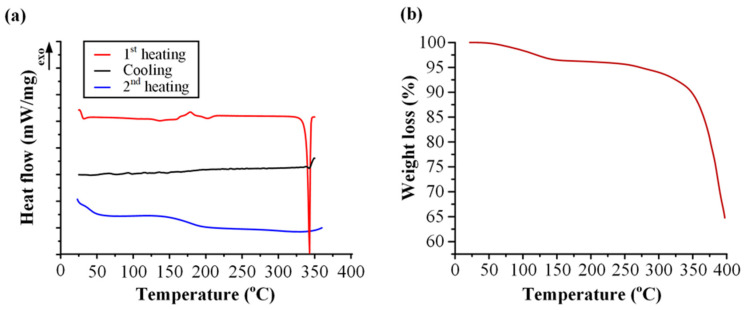
(**a**) DSC thermograms for the evaluation of GFA and (**b**) TGA thermogram for the evaluation of LUT’s thermal stability.

Figure 1a shows LUT’s DSC diffractograms used to assess its GFA. According to Baird et al. [54], the GFA of all drug compounds can be estimated by evaluating their performance in a DSC heat–cool–heat cycle. Specifically, drugs that show recrystallization during cooling from the melt are characterized as non-glass formers and belong to the class I GFA compounds. Similarly, APIs that recrystallize during DSC reheating are characterized as non-stable glass formers (GFA class II), while those that do not recrystallize, even after the second heating, are considered as stable glass formers (GFA class III). Of these, the GFA class III and II compounds are more likely to form ASDs that show good physical stability during storage, although recent studies have shown that, if properly designed, stable ASDs may also be formulated for GFA I compounds [62]. Checking the obtained results for LUT during the first heating scan, an endothermic peak was recorded at *T_m_* = 343.2 °C (ΔH_f_ = 187.5 J/g) corresponding to the melting of the drug. There were no thermal events detected during cooling, while only a single T_g_ (at 186.5 °C) was obtained after the second heating scan (reheating step). Thus, according to the obtained thermal behavior, it can be concluded that LUT is a stable glass former that belongs to the GFA class III compounds.

#### 3.1.2. Thermal Stability of LUT

In addition to GFA, the thermal stability of LUT was evaluated via TGA (Figure 1b). According to the obtained thermogram, the commercially available raw API contains approximately 3–5% of water (recorded as an initial weight loss in the TGA graph), while the API seems to be thermally stable up to approximately 300 °C. Hence, based on the above results, in the case of LUT, it would be better to avoid the use of any melt-based methods for the preparation of ASDs (such as melt mixing), since the thermal decomposition of the API is close to its melting point and there is thus a high risk of thermal degradation during the preparation of the ASDs.

### 3.2. Selection of LUT’s ASD Matrix/Carrier

Following the determination of LUT’s GFA and thermal stability profile, the ability of six commonly used polymeric matrix/carriers to inhibit LUT’s recrystallization was determined via the film casting method. Thereafter, the most promising matrix/carriers were further studied regarding their capacity to suspend LUT precipitation after solubilization (via the solvent shift method).

#### 3.2.1. Film Casting Method

The ability of the polymers to retard API’s recrystallization is demonstrated by the visual observation of binary ASD films comprised of the API and each matrix/carrier individually. Based on this film casting method, the carriers that show the most significant inhibition of the drug’s recrystallization are distinguished as the most suitable for preparing the ASDs.

**Figure 2:**
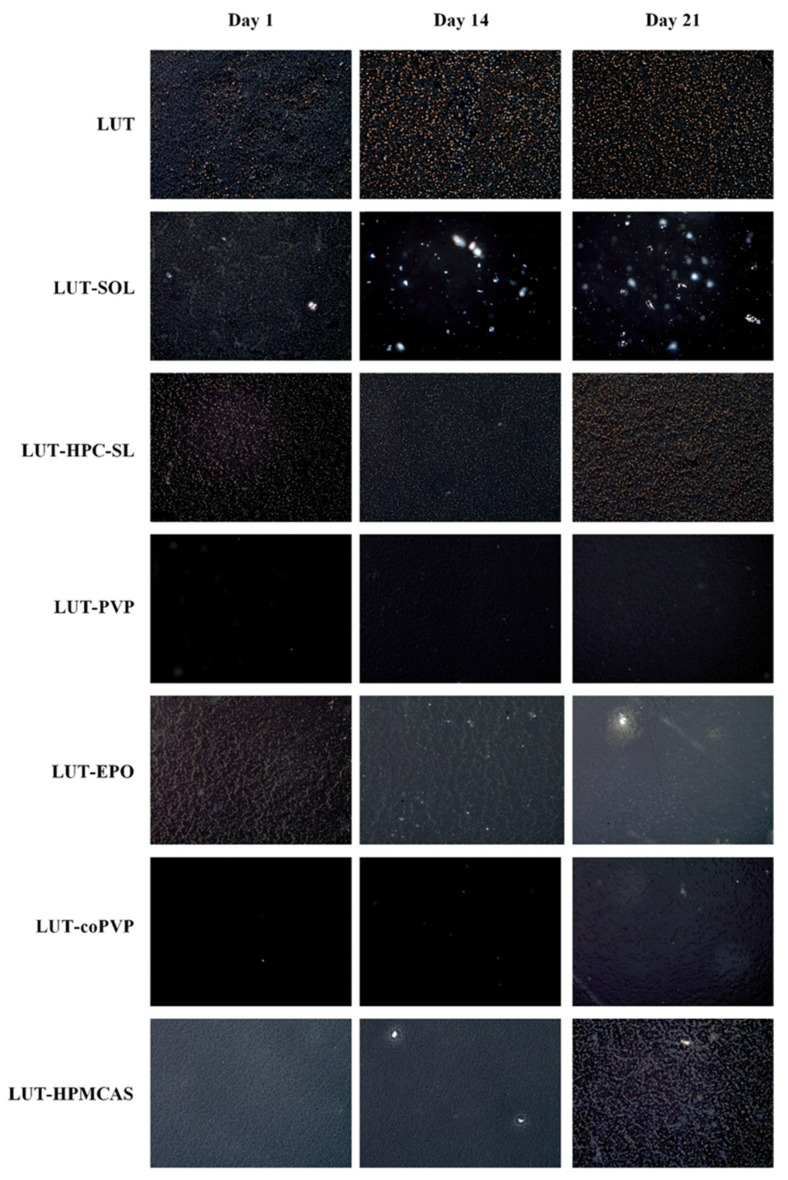
PLM photographs of neat LUT and LUT-matrix/carriers’ binary thin films after 1, 14, and 21 days of storage.

Figure 2 shows the PLM images of the prepared films for the pure LUT and the binary LUT-matrix/carriers after 1, 14, and 21 days of storage. Regarding the neat API, the obtained results showed extensive recrystallization from day 1, indicating that the API is highly prone to physical instability when it is stored in the selected conditions. Similar results were also observed when SOL, HPC-SL, and EPO were used as matrix/carriers, indicating that the use of these polymers did not also retard API’s crystal growth. Similarly, on day 14, and especially by day 21, the presence of API crystals was observed on the films prepared with HPMCAS, indicating that HPMCAS also fails to prevent API’s recrystallization during storage. On the other hand, in contrast to the rest of the tested polymers, PVP and coPVP were able to successfully suspend API’s recrystallization, while both led to good physical stability of API, even after 21 days of storage. Hence, according to the film casting method’s results, it appears that both PVP and coPVP can be considered as promising matrix/carriers for the formation of LUT ASDs.

#### 3.2.2. Solvent Shift Method

**Figure 3:**
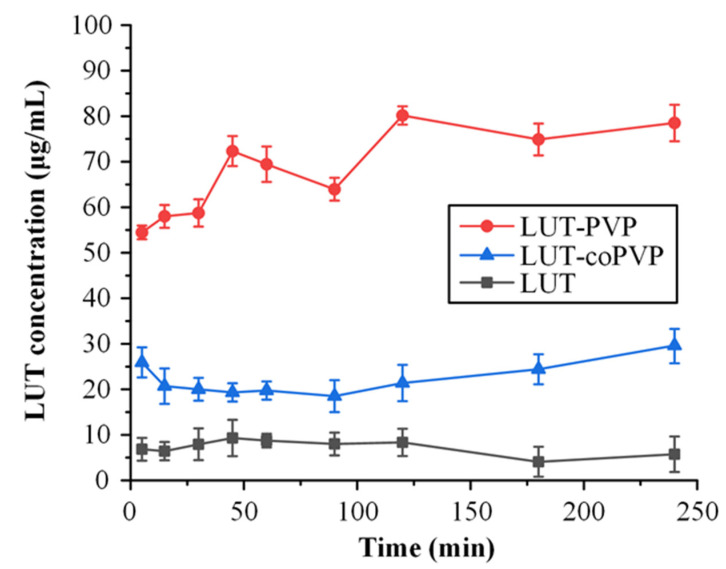
LUT concentration profile during the evaluation of matrix/carriers based on the solvent shift method.

The solvent shift method examines the ability of the matrix/carriers to inhibit API’s precipitation during solubilization and, therefore, it can be considered as an extra tool to determine the ability of ASD formulations to stabilize the APIs in the presence of the dissolution medium. In the present study, the polymers selected from the film casting method (i.e., PVP and coPVP) were further studied via the solvent shift method. Results presented in Figure 3 show that PVP outperforms coPVP in terms of precipitation inhibition, as it maintains higher levels of LUT concentration in the dissolution. Hence, although both PVP and coPVP demonstrated similar capacity regarding the inhibition of drug recrystallization in a dry state (film casting method), PVP seems to be more suitable for LUT as it prevents its precipitation in the presence of simulated saliva.

Therefore, based on both the film casting and solvent shift methods, PVP is selected as the most promising matrix/carrier for the preparation of LUT’s ASD systems suitable for the oral cavity.

### 3.3. Miscibility Evaluation

Since the selection of the most suitable matrix/carrier has been made, it is crucial to examine the miscibility of these components (i.e., LUT and PVP) before beginning with the ASD’s formulation and evaluation. In the present study, two different theoretical approaches were employed for the determination of components’ miscibility (i.e., FH interaction parameters and Hansen solubility parameters), while DSC was used to experimentally verify the suggested outcomes.

#### 3.3.1. Estimation of the Flory–Huggins (FH) Interaction Parameter

**Figure 4:**
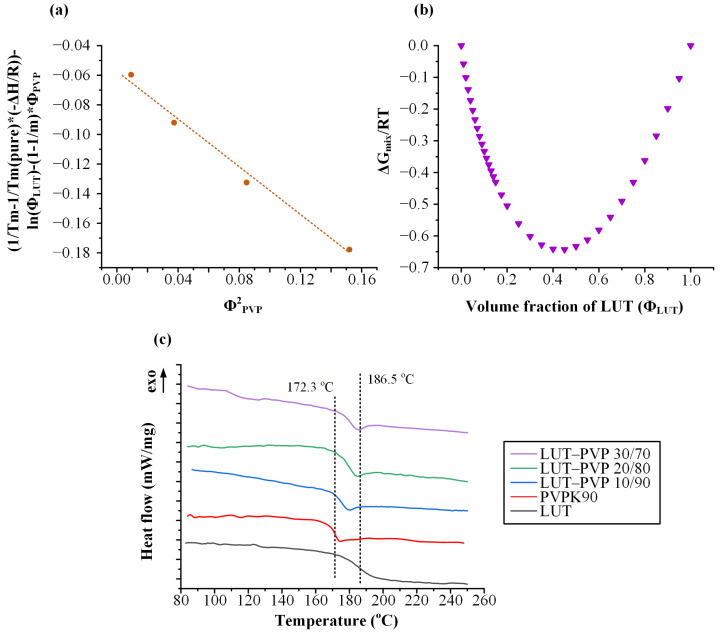
(**a**) FH interaction parameter estimation plot, (**b**) ΔG_mix_/RT vs. Φ_LUT_ plot, and (**c**) DSC depicting T_g_ values for the raw materials, and the LUT-PVP mixtures at 10, 20, and 30% *w*/*w* of API. Note: The symbol * at the picture (**a**) is used as a multiplication symbol.

According to FH lattice theory, the dispersion of any small pharmaceutical compound in a polymer may be considered as a polymer solution, with the difference that the API itself is in the place of the solvent. Hence, the calculation of the FH interaction parameter (*χ*) may consist of a thermodynamically driven factor for evaluating drug–matrix/carriers’ miscibility. In the present study, *χ* was calculated by plotting the $\left(\frac{1}{T_{m}\left(\mathrm{mix}\right)}-\frac{1}{T_{m}\left(\mathrm{LUT}\right)}\right)*\left(\frac{{\Delta H}_{\mathrm{fus}}}{-R}\right)-\ln\Phi_{\mathrm{LUT}}-\left(1-\frac{1}{m}\right)\Phi_{\mathrm{PVP}}$ factor against Φ_PVP_^2^. At low polymer *Φ*^2^ values, *χ* can be considered as independent of T and can therefore be calculated from the slope of the linear regression line. It is important to note that for the construction of the plot (Figure 4a), the melting point of LUT was experimentally determined via DSC (Figure S2, Supplementary Materials). Based on the obtained results, a negative *χ* value was calculated (*χ* = −0.844, R^2^ = 0.961), indicating that the system is thermodynamically miscible, while the negative ΔG_mix_ observed in Figure 4b with increasing PVP concentration verifies the overall efficient thermodynamic miscibility of the components independent of the polymer’s concertation.

#### 3.3.2. Hansen Solubility Parameters (HSPs)

Apart from FH lattice theory, the miscibility of the components was examined using Hansen solubility parameters derived by the Hoftyzer–Van Krevelen group contribution method. Table 1 presents the HSPs for LUT and PVP. According to the obtained results, good miscibility between LUT and the examined polymeric matrix/carrier can be assumed, given that the total solubility parameter (δ_t_) for LUT is equal to 28.65 MPa^1/2^ and the respective parameter’s value for PVP is 22.37 MPa^1/2^, leading to an absolute difference between the API’s and the polymer’s solubility parameters (Δδ_t_) of 6.28 MPa^1/2^. This finding agrees with FH interaction parameter results (Section 3.3.1), where the two compounds were found to be thermodynamically miscible.

#### 3.3.3. DSC Measurements

In addition to the FH interaction and the Hansen solubility parameters, which examine the miscibility of the two components on a theoretical basis, the miscibility of the two components was also examined experimentally with the use of DSC. Figure 4c presents the DSC thermograms (second heating scan) of the raw materials (PVPK90 and LUT) and the LUT-PVP physical mixtures at various drug-to-matrix/carrier concentrations (i.e., 10/90, 20/80, and 30/70 *w*/*w* of API to matrix/carrier). Based on the obtained results, both PVP and LUT demonstrated a single T_g_ at 172.3 °C and 186.5 °C, respectively. Similarly, in all LUT-PVP DSC thermograms, a single T_g_ was recorded (at 181.0 °C, 179.7 °C, and 177.9 °C for 10%, 20%, and 30% of LUT, respectively), proving that the two components are in a miscible state, at least in the weight fractions examined (i.e., up to 30% *w*/*w* of LUT).

### 3.4. Evaluation of LUT-PVP ASDs

After selecting the most suitable matrix/carrier (i.e., PVP) and ensuring that the two components (LUT and PVP) are miscible, the ASDs were prepared (via the solvent evaporation method) and thoroughly evaluated.

#### 3.4.1. Physical State Evaluation of ASDs

**Figure 5:**
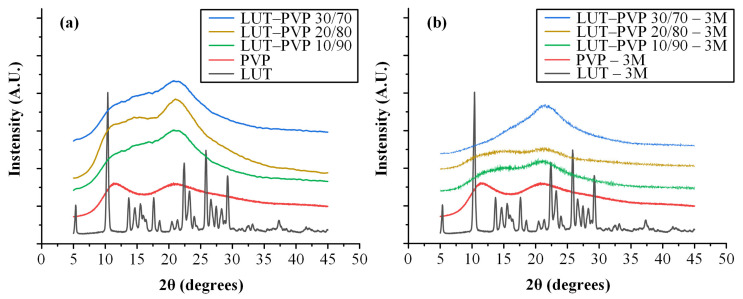
pXRD diffractograms for the raw materials (LUT and PVP) and the LUT-PVP ASDs (**a**) immediately after preparation and (**b**) after storage for 3 months (3M).

Initially, to ensure that the prepared solid dispersions were indeed amorphous, the physical state of the LUT-PVP ASDs (at different API-to-matrix/carrier-weight ratios) was examined immediately after preparation (zero-time) with the aid of the pXRD method. In Figure 5a, the obtained diffractograms for the neat LUT and PVP, as well as for the prepared ASD systems at several LUT-to-PVP-weight ratios, were recorded. The pXRD diffractogram of LUT revealed that the API is highly crystalline, showing several characteristic 2θ diffraction peaks at 5.33°, 10.36°, 13.70°, 17.62°, 22.40°, 23.46°, 23.55°, 25.84°, and 29.25°, respectively. Regarding PVP, a broad halo located at 2θ 5–45° confirmed that the polymer in its raw form is completely amorphous. Similarly, no LUT distinctive reflection peaks were found in the zero-time diffractograms for all ASDs, proving that LUT-PVP ASDs were successfully formed in all LUT weight ratios.

In addition to pXRD, DSC was also used to evaluate the physical state of the API. The DSC thermograms of the prepared ASDs (at 10/90, 20/80, and 30/70 *w*/*w* of API to matrix/carrier) compared to the pure LUT and the neat PVP (all shown in Supplementary Materials, Figure S3), prove the full amorphization of the API within the prepared systems, since no melting peaks were recorded in all cases.

Moreover, in an attempt to study LUT’s physical state during stability, the formed ASDs were stored for 3 months (3M) at 40 °C ± 5 °C and 75 ± 5% RH. According to the obtained pXRD results (Figure 5b), no significant differences were observed (compared to the zero-time patterns), demonstrating that PVP can maintain the amorphous state of the API in all ASDs, irrespective of the drug’s weight content (even at a relatively high, i.e., 30% *w*/*w*, drug loading).

#### 3.4.2. Molecular Interactions

**Figure 6:**
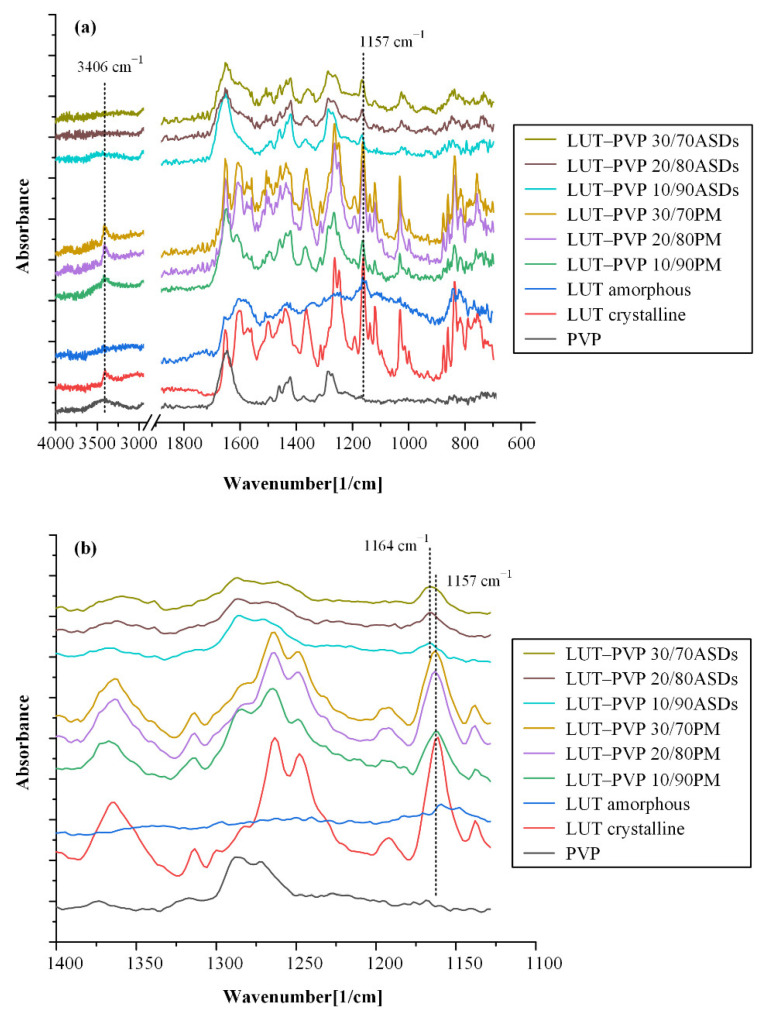
ATR-FTIR spectra of neat crystalline LUT, neat amorphous LUT, neat PVP, LUT-PVP physical mixtures (PMs), and ASDs at several drug-to-matrix/carrier-weight ratios from (**a**) 400–700 cm^−1^ and (**b**) 1400–1100 cm^−1^.

ATR-FTIR spectroscopy was utilized in an effort to determine the mechanisms responsible for the physical stability of LUT-PVP ASDs and to study the formation of molecular interactions between the API and the matrix/carrier. Figure 6 presents the ATR-FTIR spectra of raw materials (i.e., LUT and PVP), their physical mixtures (PMs), and the prepared LUT-PVP ASDs. In the case of neat API, ATR-FTIR results indicated several characteristic absorption peaks, with the most distinct being at 3406 cm^−1^ (due to O-H stretching), 1690 cm^−1^ (due to C=O symmetrical stretch), 1157 cm^−1^ (due to C-O stretching), and 1050 cm^−1^ and 830 cm^−1^ (due to C-O stretching on C-OH). Regarding the amorphous LUT, the formation of additional interactions between API’s molecules is observed, since the intensity of the characteristic absorption peak at 1690 cm^−1^ was significantly reduced, while most of the other LUT characteristic FTIR peaks (including the peak corresponding to the O-H stretching at 3406 cm^−1^) were either shifted to lower wavelengths or completely disappeared. In the case of neat PVP, results showed several characteristic peaks at 3425 cm^−1^ (corresponding to the presence of residual water), 1650 cm^−1^ (corresponding to C=O stretching), 1426 cm^−1^ (corresponding to C-H bending), and 1291 cm^−1^ (corresponding to CH_2_ wagging). Regarding the PMs, it is known that their preparation process (i.e., mild mixing in a mortar with a pestle) usually does not lead to any changes in the physical state of the mixing components and, consequently, to the formation of any new molecular interactions. This was confirmed by ATR-FTIR, where the obtained spectra for all PMs were the sum of the pure crystalline LUT and the respective pure polymeric matrix/carrier (PVP) spectra. On the contrary, the collected spectra of LUT-PVP ASDs presented significant differences in comparison to the respective PMs, indicating the formation of molecular interactions between LUT and PVP. Specifically, the absorption peak corresponding to LUT’s O-H stretching at 3406 cm^−1^ was not recorded in all ASDs spectra, indicating that the hydroxyl molecules of LUT are probably participating in the formation of new molecular interactions, either with other API molecules (i.e., intermolecular interactions), leading to the formation of neat amorphous API, or with PVP’s C=O molecules (i.e., intramolecular interactions), leading to molecularly dispersed ASDs. Moreover, the comparison of the ASDs and the neat amorphous API spectra showed that some of the LUT ATR-FTIR peaks in the ASDs were shifted (for example, the peak corresponding to LUT’s C-O stretching at 1157 cm^−1^ was shifted to 1164 cm^−1^ in all ASDs). Figure 6b indicates that drug–polymer intermolecular interactions are indeed being formed, leading thus to the formation of molecularly dispersed ASDs. These drug–polymer molecular interactions are responsible (probably partially since other mechanisms may also be involved, such as reduced molecular mobility, etc.) for the physical stability of LUT in the selected PVP-based ASDs during storage.

#### 3.4.3. MD Simulations

Computational approaches such as MD simulations are able to offer extensive atomic-level structural and energetic information that greatly aids the comprehensive evaluation of ASDs. Therefore, in the present study, MD simulations were employed, so as to further elucidate the type of molecular interactions formed within the prepared LUT-PVP ASDs.

**Preparation of simulation boxes**

**Figure 7:**
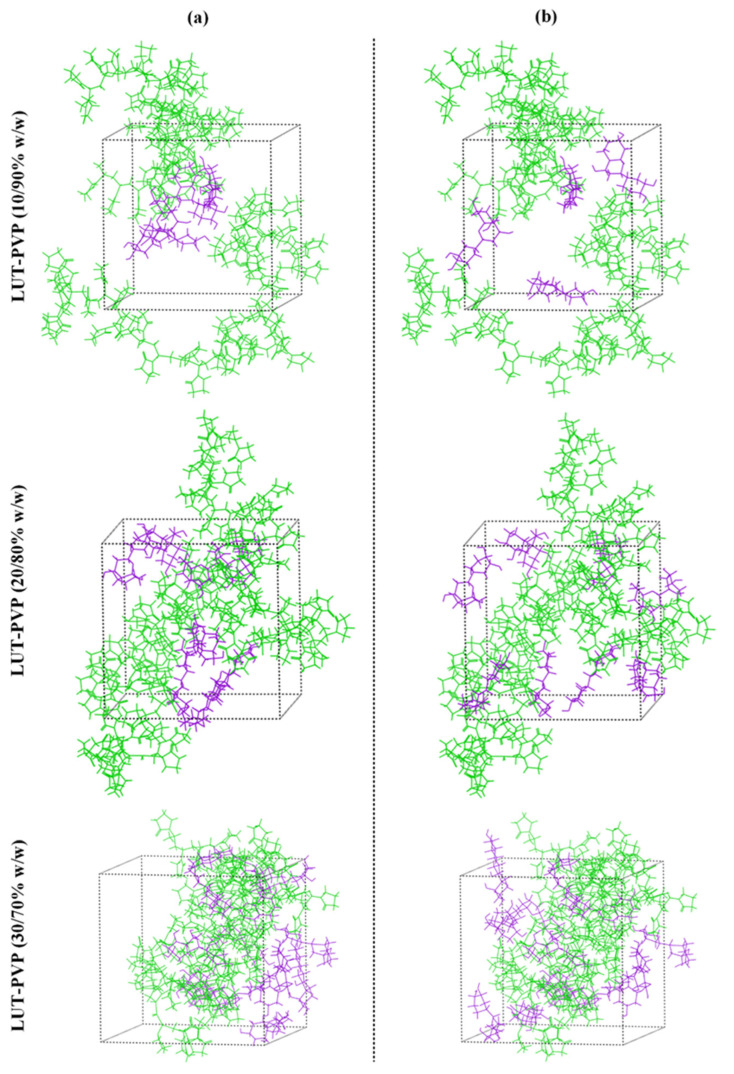
MD simulation boxes (**a**) before and (**b**) after the adopted equilibration protocol (PVP chains with green; LUT molecules with purple).

The molecular structure for the neat LUT molecule and its amorphous cell (having 20 LUT molecules), as well as the PVP chain (having 30 monomers) and its amorphous assembly (having 2 PVP chains), is seen in Figure S4 (Supplementary Materials). Regarding the LUT-PVP amorphous simulation boxes (containing 10%, 20%, and 30% *w*/*w* of API to matrix/carrier), the results in Figure 7 show that after following the suggested equilibration protocol, the final amorphous assemblies were much more homogeneous compared to those initially prepared. Hence, it can be said that the selected equilibration steps were able to prepare well-mixed molecular dispersions of the API within the selected matrix/carrier.

**Validation of amorphous assemblies**

Before continuing with the evaluation of molecular interactions, it is essential to confirm the validity of the prepared amorphous simulation boxes. This was conducted by comparing the MD- vs. DSC-derived T_g_s. In the case of MD simulations, the T_g_ values of the assemblies are estimated by plotting the averaged specific volume (*v*) against temperature during cooling. Typically, a kink (corresponding to T_g_) is recorded, which is the result of the second order-like phase transitions between the rubbery and the glassy states. For the determination of the T_g_ values, the plot is divided into glassy and rubbery state points and, then, linear regression lines are plotted for each group. The T_g_ is estimated as the point where these two regression lines converge.

In the present study, the *v* vs. *T* plots for the neat LUT and the various LUT-PVP assemblies (at several weight ratios) are shown in Figure S5 (Supplementary Materials). In all cases, the T_g_ estimated by MD (i.e., 194.8, 185.9, 188.3, and 189.6 °C for LUT, LUT-PVP 10/90%, 20/80%, and 30/70% *w*/*w*, respectively) was slightly higher compared to the actual DSC-derived values (i.e., 186.5, 176.1, 179.8, and 182.3 °C for LUT, LUT-PVP 10/90%, 20/80%, and 30/70% *w*/*w*, respectively). This can be explained by the fact that the change from the rubbery to the glassy state (i.e., the glass transition) is a time-dependent process, which, in the case of the MD simulations, occurs much faster than in DSC, thus causing an early vitrification and consequently higher T_g_s. Nevertheless, despite these differences, the MD-derived and the actual T_g_s are in good agreement, indicating that the prepared amorphous assemblies and the selected MD parameters can be deemed reliable for predicting the performance of the analyzed systems.

**Evaluation of molecular interactions**

Looking at the molecular structure of LUT (Figure S1, Supplementary Materials) the API consists of four hydrogen bond (H-bond) donors (i.e., four hydroxyl groups) and two H-bond acceptors (i.e., the 4H-1-benzopyran-4-one ketone and the ether oxygens). Similarly, PVP consists of several H-bond acceptors (i.e., the N-vinylpyrrolidone C=O groups). Hence, it is expected that, during the formation of the ASDs, the two compounds will form several H-bonds. Therefore, in the present study, the long-run MD-simulation trajectories of the various amorphous assemblies were analyzed in respect to the formation of intra and intermolecular H-bonds. For the purposes of this analysis, the H-bond maximum hydrogen-acceptor distance was set at 2.5 Å and the minimum donor hydrogen-acceptor angle was set at 90°.

**Figure 8:**
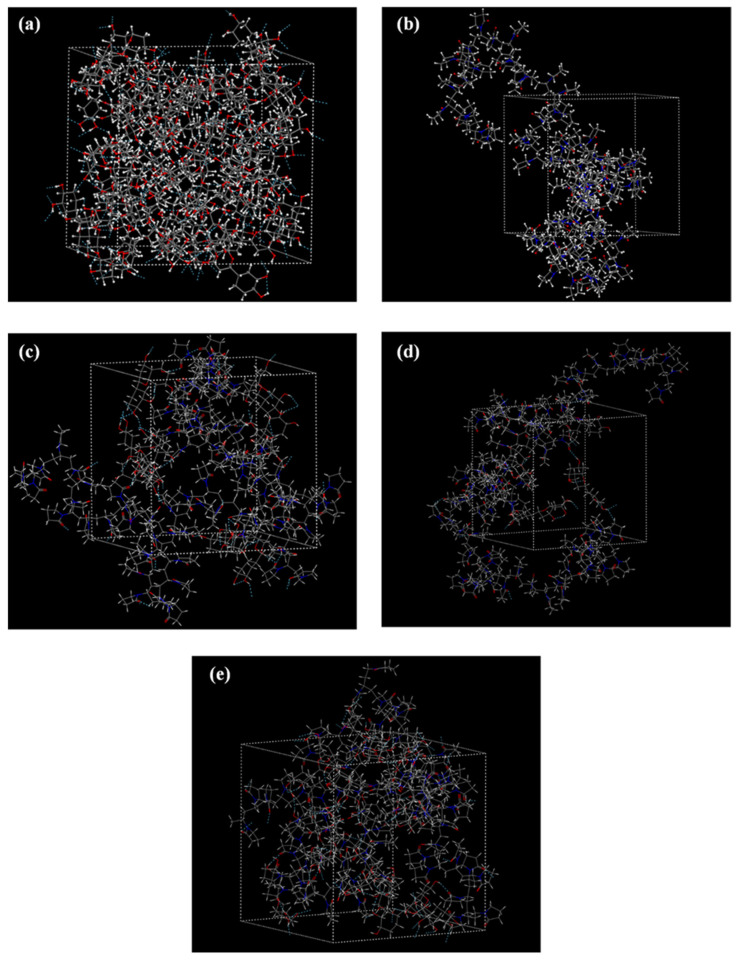
H-bond patterns in (**a**) amorphous LUT, (**b**) amorphous PVP, (**c**) LUT-PVP 10/90 *w*/*w*, (**d**) 20/80 *w*/*w*, and (**e**) 30/70 *w*/*w* ASDs. (H-bonds are depicted with cyan dashed lines.)

**Figure 9:**
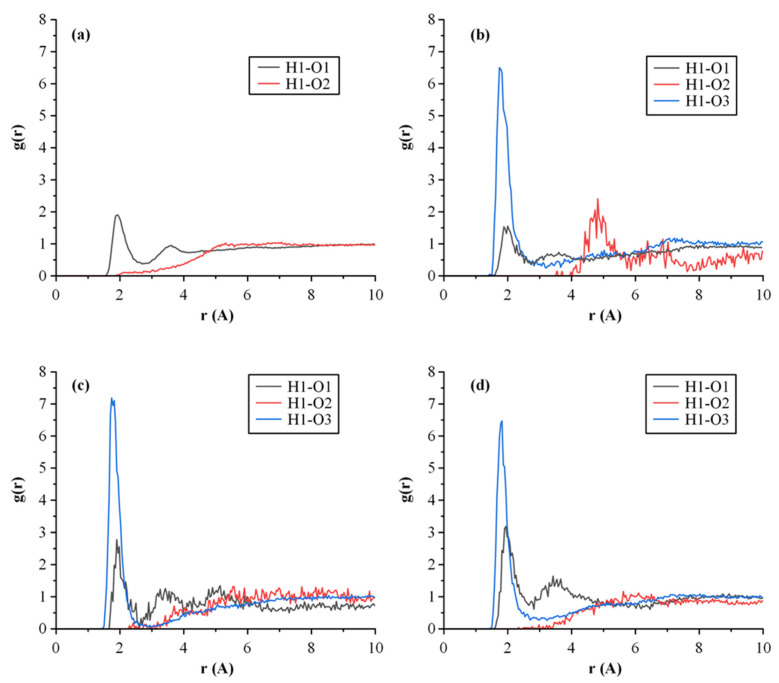
Radial distribution functions, *g*(*r*), of intermolecular interactions for the (**a**) amorphous LUT and the LUT-PVP ASDs at (**b**) 10/90, (**c**) 20/80, and (**d**) 70/30 drug-to-polymer-weight ratios. H1 = LUT’s hydroxyl protons; O1 = LUT’s hydroxyl oxygens; O2 = LUT’s ether oxygens; O3 = PVP’s ketone oxygens.

Figure 8 shows the H-bond network for the amorphous raw materials (LUT and PVP) and the LUT-PVP ASDs in several API weight ratios. In the case of neat LUT, a complex network of H-bonds was formed between the various API H-bond acceptors and donors, while the PVP neat amorphous simulation box did not show any such interactions (due to the absence of H-bond donors). On the contrary, when the API was introduced into the polymeric matrix/carrier and LUT-PVP ASDs were prepared, several drug–polymer intermolecular interactions were formed (in the form of H-bonds). Therefore, in order to elucidate the nature and strength of these interactions, a radial distribution function, *g*(*r*), was used. Figure 9 presents the *g*(*r*) graphs for LUT’s hydroxyl protons (H1) against PVP’s ketone oxygens (O3), as well as its own (intermolecular) hydroxyl and ether oxygens (O1 and O2, respectively). In general, donor-acceptor distances of 2.2–2.5 Å suggest strong interactions and distances of 2.5–3.2 Å indicate moderate interactions that are predominantly electrostatic, while distances of 3.2–4.0 Å indicate weak electrostatic interactions.

In the case of the neat amorphous LUT (Figure 9a), results proved the presence of a strong *g*(*r*) peak at 1.92 Å, demonstrating the formation of intermolecular H-bonds between API’s -OH protons and the respective -OH oxygens. This result was consistent with the ATR-FTIR findings, where the peak of the crystalline API corresponding to the O-H stretching at 3406 cm^−1^ disappeared in the ATR-FTIR spectra of the neat amorphous API. Likewise, in the case of ASDs, the presence of a new strong *g*(*r*) peak (at ~1.75 Å) between LUT’s hydroxyl protons (H1) and PVP’s ketone oxygens (O3) reveals the formation of new intermolecular interactions (i.e., H-bonds) between the API and the polymer (irrespective of LUT’s concentration). In addition, the *g*(*r*) peaks’ height corresponding to the H-bonds formed between the API molecules (i.e., H1-O1 located at ~1.92 Å) seems to increase as the API content increases. This indicates that, with increasing drug loading, the API tends to develop robust homonuclear interactions with itself leading, at some point during stability, to the formation of a distinct drug-rich amorphous region. However, based on the obtained stability results, this alleged drug-rich amorphous zone has not yet, at least for 3 months, led to any API recrystallization.

### 3.5. In Vitro Drug Release Studies in Simulated Saliva

**Figure 10:**
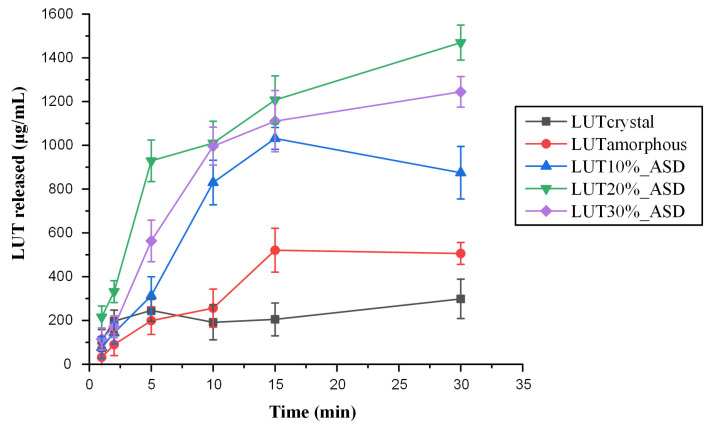
In vitro release profile of crystalline and amorphous LUT, as well as LUT-PVP ASDs at various drug concentrations.

In order to study the impact of ASDs on the solubility improvement of LUT in the oral cavity, and therefore to evaluate the efficacy of the suggested ASDs strategy to prepare suitable formulations for the topical administration of the API (either sublingually or buccally), in vitro drug release tests were conducted in simulated saliva (Figure 10). Based on the obtained results, using PVP as a matrix/carrier contributed to a significant improvement in API’s dissolution rate, compared to both crystalline and amorphous LUT. It is proven that the LUT-PVP ASD formulation increases the drug’s maximum solubility almost 6-fold compared to its crystalline counterpart and 2-fold compared to the amorphous API. This improvement was seen in all tested ASD samples, irrespective of the drug’s concentration, and can be attributed to PVP acting as a solubility enhancer, as well as a precipitation inhibitor. Therefore, based on this dissolution rate enhancement and the establishment of higher drug-dissolved concentrations in the simulated saliva (compared to the neat crystalline API), it is possible to characterize the preparation of LUT-PVP ASDs as a promising first step for the improvement of API’s solubility in the oral cavity.

## 4. Conclusions

Nowadays, the successful formulation of natural products into effective dosage forms is an ongoing challenge for the pharmaceutical industry. This is especially important in terms of complex bioactive molecules with a wide range of pharmacological properties, such as the flavonoid luteolin. In this context, the present study has been able to prepare an ASD formulation for LUT, improving its solubility in the oral cavity. Among the examined polymers and copolymers, PVP was the most appropriate matrix/carrier for the preparation of LUT ASD systems, while the prepared PVP-based ASDs proved to be stable during storage (up to 3 months). The formation and stability of the prepared ASDs can be attributed to the presence of strong intermolecular H-bonds between the API and the polymeric matrix/carrier (identified and evaluated by both ATR-FTIR spectroscopy and MD simulations). Finally, in vitro dissolution studies in simulated saliva confirmed the improvement of LUT’s solubility thanks to the use of PVP as both a solubility enhancer and precipitation inhibitor. In fact, it was shown that the examined LUT-PVP ASDs were able to increase API’s solubility by almost 6-fold in comparison with the neat crystalline drug. Thus, it can be concluded that the development of LUT-PVP ASD, administrated in a sublingual/buccal or topical (in mouth) route, could be a promising approach for the incorporation of LUT in the treatment of many severe mouth illnesses, including periodontal disease.

## Figures and Tables

**Table 1:** Estimation of HSPs for LUT and PVP based on the Hoftyzer–Van Krevelen group contribution method.

Structural Group	N	N·Fdi (J^1/2^ cm^3/2^ mol^−1/2^)	N·Fpi 2 (J cm^3^ mol^−2^)	N·Ehi (J mol^−1^)
LUT				
–OH	4	840	1,000,000	80,000
=CH–	3	600	0	0
–CO–	1	290	592,900	2000
–O–	1	100	160,000	3000
=C<	5	350	0	0
Substituted Benzyl Ring	1	1270	12,100	0
Sum		3450	1,765,000	85,000
	**δ_t_ (MPa^1/2^)**	***δ_d_*** **(MPa^1/2^)**	***δ*** **_p_** **(MPa^1/2^)**	***δ_h_*** **(MPa^1/2^)**
	28.65	18.08	7.00	21.11
**PVP**				
–CH2–	4	1080	0	0
>CH–	1	80	0	0
>N–	1	20	640,000	5000
–CO–	1	290	592,900	2000
Sum		1470	1,232,900	7000
	**δ_t_ (MPa^1/2^)**	***δ_d_*** **(MPa^1/2^)**	***δ*** **_p_** **(MPa^1/2^)**	***δ_h_*** **(MPa^1/2^)**
	22.37	16.40	12.38	8.83

## Data Availability

The data presented in this study are available on request from the corresponding author.

## Supplementary Materials

The following supporting information can be downloaded at: www.mdpi.com/article/10.3390/polym15010169/s1, Figure S1: Structural formula of LUT.; Figure S2: DSC thermograms of LUT-PVP binary mixtures (first heating scan), used for the determination of FH parameter (χ).; Figure S3: DSC thermograms of neat LUT, neat PVP, and LUT-PVP ASDs at weight ratios of 10, 20, and 30 % *w*/*w* of LUT to PVP.; Figure S4: Chemical structures of LUT (a), MD-simulation box containing 20 LUT molecules (b), 20-monomer PVP chain (c), and MD-simulation box containing two amorphous 20-monomer chains of PVP (d) (hydrogen atoms with white, carbon atoms with grey, oxygen atoms with red, and nitrogen atoms with blue).; Figure S5: Specific volume (v) vs. temperature for neat LUT (a) and LUT-PVP 10% (b), 20% (c), and 30% (d) w/w LUT (values corresponding to the glassy state are depicted with-□–and the rubbery state with-■-. Values that were not used in the analysis are shown with–x–).
