# Supplementary material for: Preparation and Evaluation of Amorphous Solid Dispersions for Enhancing Luteolin’s Solubility in Simulated Saliva
Source: Polymers (Basel). 2022 Dec 29;15(1):169. doi: 10.3390/polym15010169 (PMC9824002; doi:10.3390/polym15010169)
Supplement: Supplementary file 1 [file polymers-15-00169-s001.zip › polymers-2104940-supplementary.pdf]

Supplementary Materials

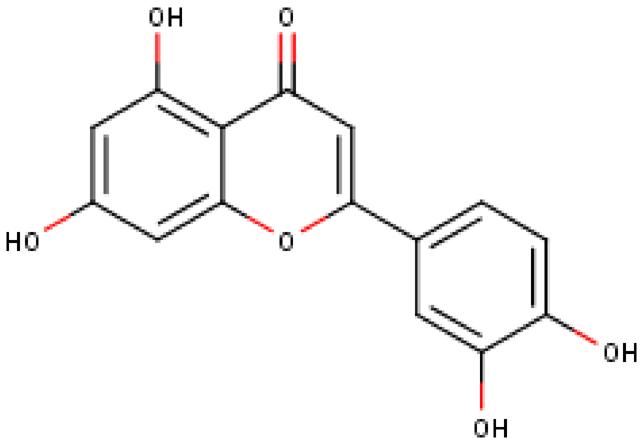

Figure S1. Structural formula of LUT.

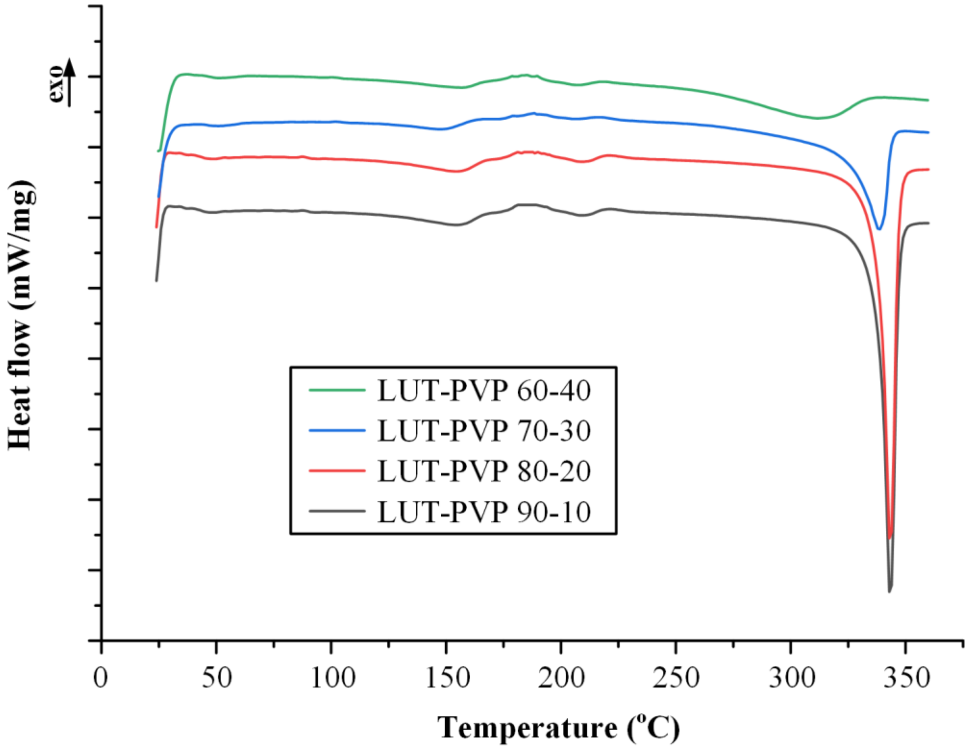

Figure S2. DSC thermograms of LUT-PVP binary mixtures (1st heating scan), used for the determination of FH parameter (χ).

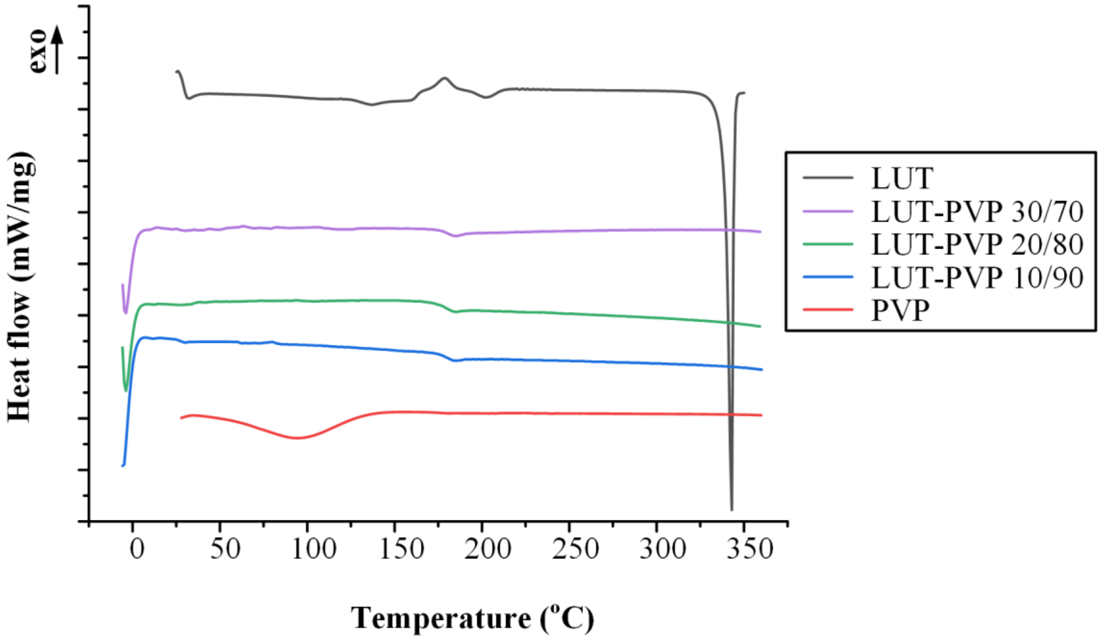

Figure S3. DSC thermograms of neat LUT, near PVP and LUT-PVP ASDs at weight ratios of 10, 20 and 30 % w/w of LUT to PVP.

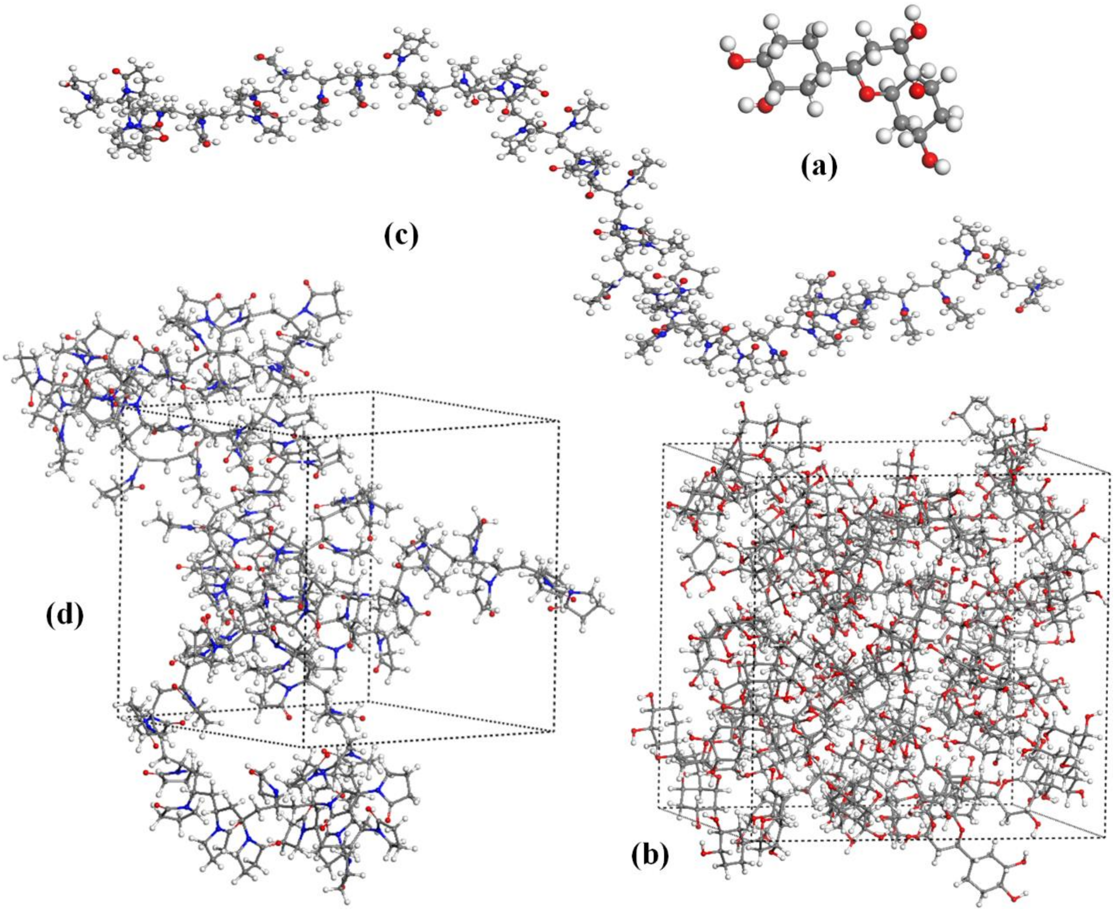

Figure S4. Chemical structures of LUT (a), MD-simulation box containing 20 LUT molecules (b) 20-monomer PVP chain (c), and MD-simulation box containing two amorphous 20-monomer chains of PVP (d) (hydrogen atoms with white, carbon atoms with grey, oxygen atoms with red and nitrogen atoms with blue).

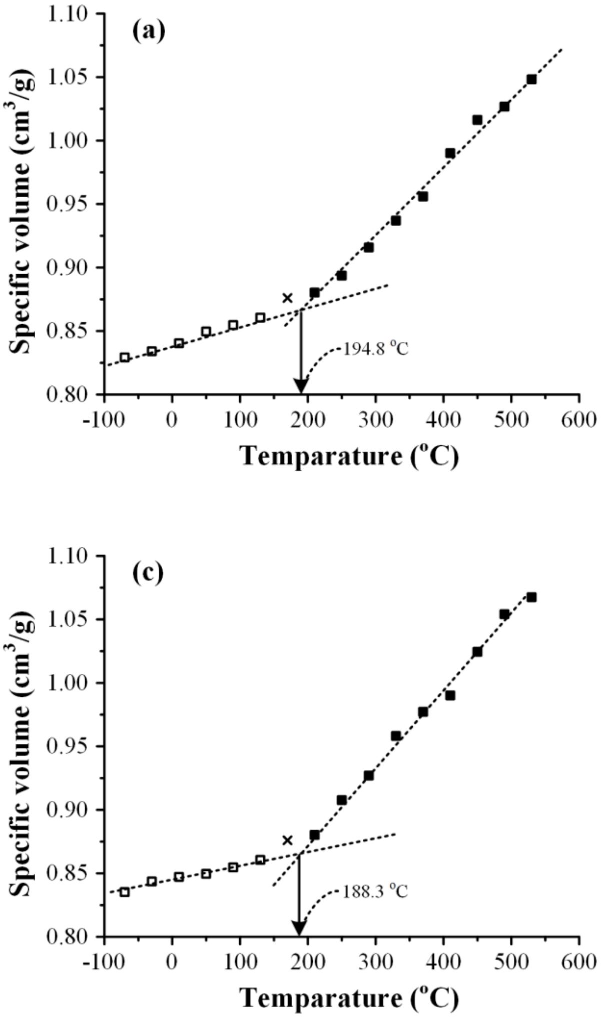

Figure S5. Specific volume (*v*) *vs.* temperature for neat LUT (a) and LUT-PVP 10% (b), 20% (c) and 30% (d) w/w LUT (values corresponding to the glassy state are depicted with - □ – and the rubbery state with - ■ -. Values that were not used in the analysis were shown with – x –).
